# Supplementary material for: Empirical methods for controlling false positives and estimating confidence in ChIP-Seq peaks
Source: BMC Bioinformatics. 2008 Dec 5;9:523. doi: 10.1186/1471-2105-9-523 (PMC2628906; doi:10.1186/1471-2105-9-523)
Supplement: Additional File 1 — A variety of html documents from the USeq web site detailing the available applications, their best usage, output file type descriptions, command line menus, etc. [file 1471-2105-9-523-S1.zip › USeqUserGuides/JavaDoc.zip!/JavaDoc/trans/main/package-use.html]

Uses of Package trans.main


|  |  |  |
| --- | --- | --- |
| **Overview** \| **Package** \| Class \| **Use** \| **Tree** \| **Deprecated** \| **Index** \| **Help** | |  |
| PREV   NEXT | **FRAMES**    **NO FRAMES**     **All Classes** |  |


---


## **Uses of Package trans.main**

| Packages that use trans.main | |
| --- | --- |
| **trans.anno** | For associating intervals, binding regions, and binding peaks with GFF annotation. |
| **trans.graphics** | For graphically representing '.cel' files and intervals. |
| **trans.main** | Principle classes and scripts used in TiMAT2 analysis. |
| **trans.misc** | Rough working scripts. |

Classes in trans.main used by trans.anno

****Interval****
  
          Container for holding information about a potential enriched region: windows, oligos, sub windows, binding peaks....

****Window****
  
          Class to hold information about a window, a defined portion of the genome typically < 1kb.

Classes in trans.main used by trans.graphics

****Interval****
  
          Container for holding information about a potential enriched region: windows, oligos, sub windows, binding peaks....

****Oligo****
  
          Container to hold information about a particular oligo.

Classes in trans.main used by trans.main

****BindingPeak****
  
          Holds information about a binding peak within an interval.

****Interval****
  
          Container for holding information about a potential enriched region: windows, oligos, sub windows, binding peaks....

****Oligo****
  
          Container to hold information about a particular oligo.

****PosRegion****
  
          For holding information about a genomic interval, what genes are up, down, overlap, or contain this genomic inteval.

****SubWindow****
  
          Class to hold information about a subWindow.

****WilcoxonSamplePair****
  
          Used by the `WilcoxonSignedRankTest`.

****Window****
  
          Class to hold information about a window, a defined portion of the genome typically < 1kb.

Classes in trans.main used by trans.misc

****Interval****
  
          Container for holding information about a potential enriched region: windows, oligos, sub windows, binding peaks....

****Window****
  
          Class to hold information about a window, a defined portion of the genome typically < 1kb.

---


|  |  |  |
| --- | --- | --- |
| **Overview** \| **Package** \| Class \| **Use** \| **Tree** \| **Deprecated** \| **Index** \| **Help** | |  |
| PREV   NEXT | **FRAMES**    **NO FRAMES**     **All Classes** |  |


---
